# Supplementary material for: Assessment of practices and barriers toward nasogastric tube rehydration for moderate and severe dehydration due to diarrheal disease in under-five children among health centers in Gamo Zone, Ethiopia
Source: PLoS One. 2026 Feb 19;21(2):e0343208. doi: 10.1371/journal.pone.0343208 (PMC12919787; doi:10.1371/journal.pone.0343208)
Supplement: Attachment — Submitted filename: Response to Reviewers.docx [file pone.0343208.s002.docx]

Reviewer's Responses to Questions

**Comments to the Author**

1. Is the manuscript technically sound, and do the data support the conclusions?

The manuscript must describe a technically sound piece of scientific research with data that supports the conclusions. Experiments must have been conducted rigorously, with appropriate controls, replication, and sample sizes. The conclusions must be drawn appropriately based on the data presented.

Reviewer #1: Partly

2. Has the statistical analysis been performed appropriately and rigorously?

Reviewer #1: Yes

3. Have the authors made all data underlying the findings in their manuscript fully available?

The [PLOS Data policy](http://track.editorialmanager.com/CL0/http:%2F%2Fwww.plosone.org%2Fstatic%2Fpolicies.action%23sharing/1/010f019a8fc68686-6add2d9d-b187-431d-95ec-888b9c1eb469-000000/ktXgSS2qPK9KD-DsLTKdNXtuoYgwcjmuL6JBsS72Qww=237) requires authors to make all data underlying the findings described in their manuscript fully available without restriction, with rare exception (please refer to the Data Availability Statement in the manuscript PDF file). The data should be provided as part of the manuscript or its supporting information, or deposited to a public repository. For example, in addition to summary statistics, the data points behind means, medians and variance measures should be available. If there are restrictions on publicly sharing data—e.g. participant privacy or use of data from a third party—those must be specified.

Reviewer #1: Yes

4. Is the manuscript presented in an intelligible fashion and written in standard English?

PLOS ONE does not copyedit accepted manuscripts, so the language in submitted articles must be clear, correct, and unambiguous. Any typographical or grammatical errors should be corrected at revision, so please note any specific errors here.

Reviewer #1: No

5. Review Comments to the Author

Please use the space provided to explain your answers to the questions above. You may also include additional comments for the author, including concerns about dual publication, research ethics, or publication ethics. (Please upload your review as an attachment if it exceeds 20,000 characters)

Reviewer #1: The authors do a good job of identifying a gap in clinical practice and evaluating the current practice, attitudes, and barriers, particularly in remote areas. The manuscript needs further clarification in areas and significant editing for grammar and spelling. Please see further details in the comments below:

Abstract:

Conclusion (line 40-42) - presents new data regarding challenges to use of nasogastric tube. Conclusion should just summarize study results and next steps. The data related to challenges in the conclusion should be presented in the results.

- Thank you, we have fixed it.

Introduction:
Line 55 – Please write out Nasogastric tube (NGT) or any abbreviations with the abbreviation in parentheses the first time it appears in the manuscript so that it isn’t confusing to the reader. Then you can just use the abbreviation in the remainder of the manuscript.

- Thank you, we have corrected it.

Editing for English grammar and language would be helpful for readability. For example, Line 57-58 states “this reliance on IV therapy could lead to out off any treatment options risks children’s lives, as the inability to secure an IV line can delay life-saving interventions.” – I’m not sure what “out off” means. Is this supposed to be “cut off”?

- Thank you “out off” Is spelling error. We wanted to say “out of” implying “without”. We have replaced the statement with *“this reliance on IV therapy could leave clinicians without any treatment options*”

Line 59 stating that experienced clinicians are always promoted to urban areas seems very definitive. Do you have a reference for this? If not, I would just state that commonly, experience clinicians are promoted to urban areas.

- Thank you, we have corrected it.

For lines 60-64, do you have any references that may support these thoughts? Or other studies that have found these same challenges? Try to provide details related to what is known and or gaps in the literature to better introduce the reason for your study.

- Thank you, we have made some modification based on your comment.

Methods:
Data Collection, line 100-101: for alternate therapy (either nasogastric tube) is incomplete thought. Are there other alternate therapy options that were accepted?

- Thank you for the comment
  - Practicing alternative therapies such as intraosseous resuscitation and venous cut-down is clearly prohibited in health centers. The treatment protocol recommends referral; if referral is not possible, then IV resuscitation, and if that is not feasible, nasogastric tube (NGT) resuscitation.
  - We have rewritten it this way:

“*If the participant chose to try either oral fluids or placing an intravenous(IV) line, they were informed that this method was unsuccessful and that the nearby hospital is more than 30-minute drive away. This continued until the participants chose an alternative therapy, either NGT, or persisted with their original choice (IV, ORS, or referral) after being reminded three times”*

Results:
Further description of all the themes would be helpful under attitudes and challenges, potentially in a table with number of times it was mentioned would be helpful. There are only 3 listed in the attitudes table and several in the challenges table; however, some are not well understood.

- Thank you, we have made some improvements.
  - We have replaced the figure that describe Challenges in managing dehydration with a table (Table 2) and added a new table for the barriers to wards using NGT (Table 3).

Chart with attitudes –Were there any negative attitudes?

- Thank you, there weren’t negative attitudes. All of them expressed the importance of NGT in salvaging children’s lives. Though some of the participants were cautious about the complications. We have now added a few codes that imply a positive attitude (Table 1) and presented their concern about the complications in words.

Chart with Challenges in managing Dehydration – there are several misspelled words. Additionally, not sure what desrespection is – can you please define somewhere or consider another word.

- Thank you, Desrepection is misspelling for “disrespecting”. We have now replaced it with “disrespecting patients”. And we have corrected other spelling errors.

Discussion
There continue to be several errors in grammar and spelling throughout.
Line 188 has a misspelling (sing instead of sign).
Line 213 has a misspelling (challeng instead of challenge)

- Thank you, we have corrected the spelling and grammatical errors.
