# Supplementary material for: Assessment of practices and barriers toward nasogastric tube rehydration for moderate and severe dehydration due to diarrheal disease in under-five children among health centers in Gamo Zone, Ethiopia
Source: PLoS One. 2026 Feb 19;21(2):e0343208. doi: 10.1371/journal.pone.0343208 (PMC12919787; doi:10.1371/journal.pone.0343208)
Supplement: Attachment — Submitted filename: Response_to_Reviewers_auresp_2.docx [file pone.0343208.s003.docx]

**ACADEMIC EDITOR:**

The authors discuss "Assessment of practice and barriers towards nasogastric tube rehydration of moderate and severe dehydration due to diarrheal disease in under-five children among health centers in Gamo zone, Ethiopia." The manuscript requires minor improvement in several areas. I recommend its acceptance for publication following minor revisions. The key concerns are outlined below:

1. Line 181: Check grammatical errors in the line.

**Response: Thank you for your comment. We have corrected the grammatical and spelling errors in this line.**

2. Table 2: Please check whether it is ‘Themes’ or ‘Theme.’

**Response: Thank you. The heading has been corrected to ‘Theme.’**

3. Please always leave a space between the number and SI unit, no space before the "%", "/", and ":" signs. Please check all units in the whole text. There are inconsistencies in the expression and format of units.

**Response: Thank you for this important observation. We have checked the entire manuscript for spacing, consistency and units format and corrected the errors.**

4. Lastly, the text should be reviewed for grammatical, formatting, and punctuation errors. It is advisable to seek the assistance of a native English speaker to revise and proofread the manuscript before resubmission.

**Response: We have thoroughly reviewed the manuscript for grammatical, formatting and punctuation errors and identified issues have been corrected.**
